# Parliament and the Professions

**Published:** 1919-04-26

**Authors:** 


### PARLIAMENT AND THE PROFESSIONS.

### District Nurses.

Dr. Addison stated that he is well aware of the valuable work done by district nurses throughout the country, and that several district nursing associations in Lancashire and Cheshire are already co-operating with the local authorities in providing midwifery and nursing services. The Local Government Board will continue to encourage such co-operation.

### Overseas Nurses.

In reply to a question by Lieutenant-Colonel Campion, Mr. Forster stated that the valuable work done in British hospitals by nurses who came to the United Kingdom from the Dominions voluntarily and at their own expense was highly appreciated. Those who have become members of the nursing branches under the direct control and administration of the War Office have already been provided for in the way of repatriation, and it has recently been arranged to repatriate at the cost of public funds members of certain organisations, not under the direct control of the War Office, whose services may have been utilised in military hospitals.

### Tetanus and the British Troops.

Mr. Churchill stated that the number of cases and deaths from tetanus amongst the wounded arriving in this country are as follows:

| | No. of cases. | No. of deaths. |
|---|---|---|
| 1914 | 192 | 104 |
| 1915 | 134 | 75 |
| 1916 | 501 | 182 |
| 1917 | 353 | 68 |
| 1918 | 266 | 68 |

